# Skeletal muscle assessment to understand cardiometabolic interactions

**DOI:** 10.1186/1532-429X-18-S1-P220

**Published:** 2016-01-27

**Authors:** Vidhya Kumar, Henry Chang, Suzanne Smart, Beth McCarthy, Ning Jin, Subha V Raman

**Affiliations:** 1 grid.261331.40000000122857943Ohio State University, Columbus, OH USA; 2 Siemens Medical Solutions, Columbus, OH USA

## Background

Patients with diabetes and metabolic disorders have excess mortality after myocardial infarction (MI). Their mitochondrial function is often abnormal, and can be measured with phosphorus magnetic resonance spectroscopy (PMRS). Participation in a program of cardiac rehabilitation and secondary prevention (CRSP) reduces post-MI mortality, but typically involves only aerobic exercise and may not sufficiently improve mitochondrial function. An integrated assessment of skeletal muscle would potentially be useful to assess the impact of aerobic plus resistive exercise post-MI.

## Methods

We tested a combined MR-based protocol with: 1) PMRS of quadriceps muscle at rest, during 30s of isometric quadriceps exercise, and during recovery and 2) quadriceps muscle fat quantification using a multi-echo Dixon sequence at 1.5 Tesla (Siemens, Erlangen). After shimming, an unlocalized FID sequence using the following parameters was used to acquire ^31^P spectra: TR = 1000 ms, TE = 0.34 ms, BW = 2000 Hz, points = 1024, averages = 4. Fat/water quantification was acquired with: TR = 11.1 ms, 6 echoes with TE minimized, BW = 1150 Hz, slice thickness = 4 mm.

**Figure 1:**
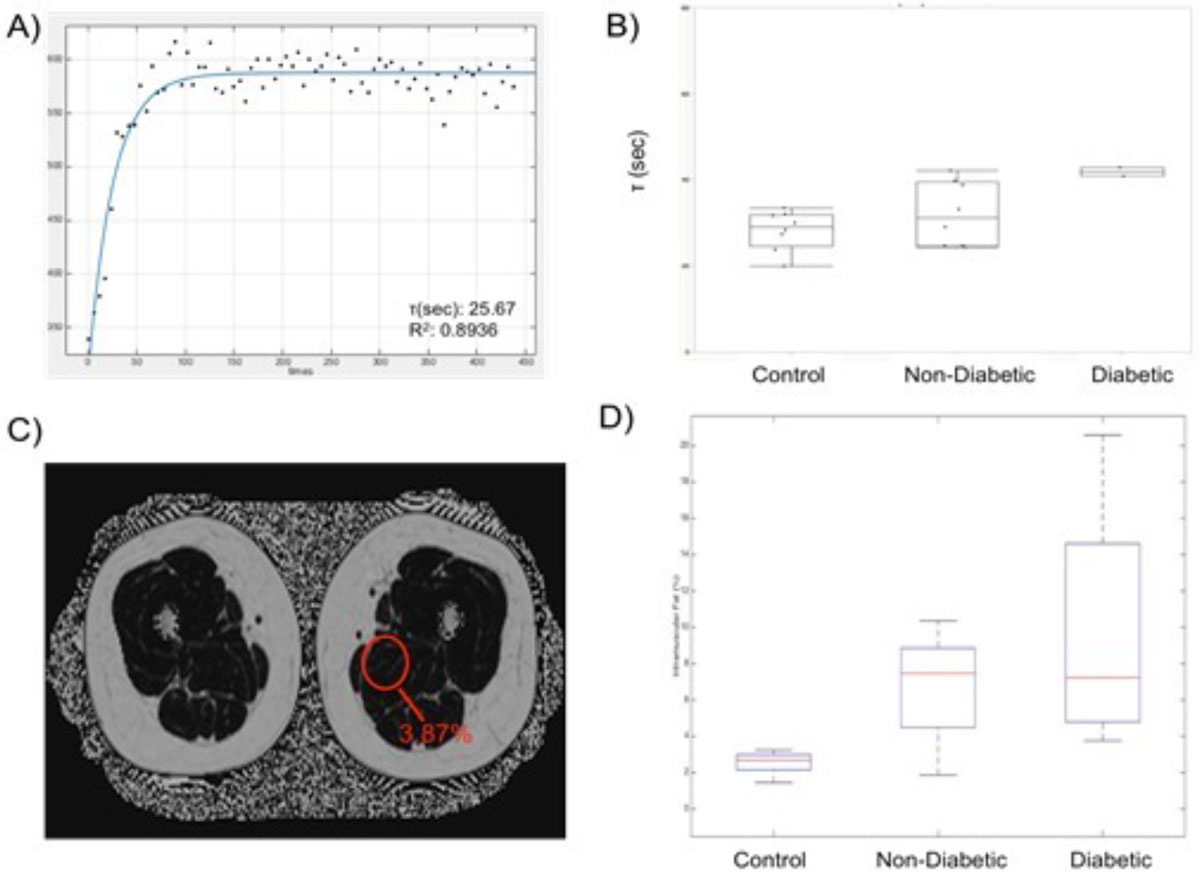
**A) Phosphocreatine (PCr) concentration is depleted with rapid resistive extremity exercise and returns to baseline levels within a recovery period (τ), which is a well-established biomarker of mitochondrial oxidative function**. A representative recovery curve is shown. B) Comparison of PCr recovery times demonstrates sequentially poorer mitochondrial oxidative capacity in control subjects, non-diabetic and diabetic patients. C) A representative quantitative fat image acquired through the leg illustrates the measurement of intramuscular lipid. D) Comparison of results indicates sequentially higher skeletal muscle fat content in control subjects, non-diabetic and diabetic patients.

PCr peak amplitudes, representing concentration, were quantified using jMRUI (Lyons, France) and recovery time was calculated with a best-fit mono-exponential function (**Fig** 1A). Quantitative fat maps were generated from the Dixon sequence, with pixel intensity representing fat percentage (**Fig** 1C). Feasibility was assessed in healthy volunteers and patients starting a CRSP program post-MI.

## Results

Nine volunteers and 15 patients were enrolled. Left ventricular ejection fraction was preserved in CRSP patients (56 ± 10%). Maximum exertion ability, measured before starting CRSP was similar in diabetic and non-diabetic patients (3.05 ± 0.6 vs. 3.4 ± 0.8 metabolic equivalents [METs], p = 0.4). Hba1c averaged 7.8% in diabetics whose LDL levels averaged 109.7 ± 51.5 vs. 106.7 ± 33.4 mg/dL in nondiabetics (p=0.9). PCr recovery time was longer (41.9 ± 1.4 vs. 32.1 ± 7.4 s, p = 0.05), and intramuscular fat percentage higher in CRSP patients vs. controls (8.7 ± 2.9 vs. 2.54 ± 0.6%, p < 0.001) (**Fig** 1B,D). Intramuscular fat percentage was similar in diabetic and non-diabetic patients prior to starting CRSP (p =0.4), and PCr recovery time tended to be longer in diabetic patients compared to nondiabetic patients and controls (p=0.03 for trends across groups). Preliminary follow-up data suggest considerably worse improvement in METs in diabetic vs. nondiabetic patients (delta = 1.0 ± 0.8 vs. 4.0 ± 2.4, p = 0.06).

## Conclusions

An integrated protocol of skeletal muscle 31P spectroscopy with fat quantification is feasible in patients starting cardiac rehabilitation, and may help improve understanding of cardiometabolic interactions that are not evident from cardiac measures alone.

